# In-field estimation of vertical distribution of total nitrogen and nicotine content for tobacco plants based on multispectral and texture feature fusion

**DOI:** 10.3389/fpls.2025.1647566

**Published:** 2025-10-23

**Authors:** Wenwu Liu, Weimin Guo, Junying Li, Yanling Zhang, Hanping Zhou, Aiguo Wang, Yuxin Hou, Qi Guo, Qiang Xu, Xuan Song

**Affiliations:** ^1^ Zhengzhou Tobacco Research Institute, China National Tobacco Corporation, Zhengzhou, China; ^2^ Pingdingshan Branch of Henan Provincial Tobacco Company, Pingdingshan, China; ^3^ School of Cyber Science and Engineering, Zhengzhou University, Zhengzhou, China

**Keywords:** tobacco leaf segmentation, multispectral, texture features, total nitrogen, nicotine

## Abstract

Accurately obtaining the total nitrogen and nicotine content of tobacco plants and their vertical distribution within the canopy is crucial for smart management and quality assessment. However, the complex field environment and uneven vertical distribution pose significant challenges for precise estimation. This study proposed a spectral and texture feature fusion method based on deep learning to improve estimation accuracy, and an improved YOLOv8 model (AO-YOLOv8) was developed for tobacco leaf instance segmentation. After segmentation, the average spectral features from six image channels were extracted, and 474 texture features were obtained using Gray Level Co-occurrence Matrix (GLCM), Local Binary Pattern (LBP), Fourier Transform, Gabor Filter, and Wavelet Transform. Four deep neural networks, including LSTM, RNN, MLP, and FCNN, were then applied to establish estimation models of nitrogen and nicotine content at both the leaf and plant scales. The results showed that AO-YOLOv8 achieved an mAP50 of 87.3 and an mIoU of 83.4 in the leaf instance segmentation task, representing improvements of 6.99% and 8.88% over the original YOLOv8, and effectively detected and separated overlapping leaves under complex conditions. The fusion of spectral and texture features significantly improved prediction accuracy, with the LSTM network achieving the best performance, yielding R^2^ values of 0.8634 and 0.8735 for nitrogen and nicotine prediction at the leaf scale in laboratory conditions. In the field environment, the LSTM-based models for plant-scale nitrogen and nicotine estimation achieved R^2^ values of 0.6771 and 0.5735, respectively, which outperformed models using spectral features alone. In conclusion, this study enabled accurate estimation and visualization of the vertical distribution of nitrogen and nicotine content in field-grown tobacco plants, providing an efficient, low-cost, and non-destructive solution for tobacco production and quality control.

## 1 Introduction

Nitrogen is an essential element that significantly influences the growth and development of tobacco (Collins and Hawks, 1993). It is also an important component of nicotine and has a significant impact on the synthesis and accumulation of nicotine (MacKown and Sutton, 1997). Nicotine is a critical factor in determining the flavor of tobacco, greatly affecting the quality of tobacco leaves and the final product (Zhang et al., 2024). Compared to reducing sugars and proteins, nitrogen and nicotine content undergo relatively small changes before and after the curing process (Chen et al., 2021a). Therefore, timely estimation of nitrogen and nicotine content in field-grown tobacco is not only beneficial for precise implementation of field management practices, such as fertilization, but also has an important significance for the prediction of tobacco leaf quality after roasting and the allocation of industrial enterprises.

Traditional methods for detecting the chemical compositions of tobacco leaves, including chemical and laboratory analyses, often require large quantities of time and high costs. These methods are limited by sampling representativeness and operational complexity accompanied with the disadvantage of result lag (Simonne et al., 1997; Tang et al., 2019). With the development of smart agriculture technologies, spectral analysis has gradually become an important tool in plant research due to its non-destructive, rapid, and efficient characteristics. Multispectral data, especially in the visible and near-infrared range, can provide valuable information about the chemical composition of plants. Some studies have utilized the canopy spectra of tobacco fields, combined with first-order derivative spectra, vegetation index and hyperspectral parameters, and adopted multiple linear regression to establish a prediction model. The characteristics highly correlated with nitrogen content were studied and analyzed. The model verification results reached R²=0.73 and RMSE=0.38, which can accurately predict the nitrogen content of tobacco leaves (Guo et al., 2023). In terms of nicotine content prediction, existing studies have shown that a prediction model can be constructed by combining ultraviolet spectral data with multiple regression methods, and there is a significant correlation between the predicted values and the true values. Furthermore, some scholars have adopted hyperspectral imaging techniques to extract the average spectra of tobacco samples and conducted modeling studies by combining multiple regression algorithms such as PLSR, SVR, RF, and PLSR-VIP. Among them, the PLSR model performs best throughout the entire band range (R²=0.93). The PLSR-VIP model can maintain a relatively high prediction accuracy (R²=0.91) even when using only five key bands (Wei et al., 2018; Divyanth et al., 2022). There are also studies applying NIR and MIR spectral fusion to rapidly predict total nicotine, total sugar, reducing sugar, and total nitrogen in tobacco. By combining variable selection with multiple algorithms, these fusion approaches improve prediction accuracy compared with single-spectrum models, demonstrating the potential of spectral fusion for non-destructive chemical analysis of tobacco leaves (Wang et al., 2024a). Beyond spectral features, texture information has been proven to be effective in capturing fine-grained variations in leaf surface morphology, which contributes to quality evaluation tasks. Color and texture features extracted during different curing stages have been utilized to develop a highly accurate moisture content prediction model, achieving an R² of 0.9987 (Chen et al., 2021b). Therefore, we aim to extract the sensitive features from multispectral images to estimate the total nitrogen and nicotine content of tobacco plants rapidly.

Current methods are generally limited to canopy-level observations and lack the capacity to capture biochemical information from the middle and lower leaf positions. To obtain the biochemical information of the middle and lower leaves, the segmentation of the leaves is the first problem to be solved. Liu et al. (2020) applied the YOLOv3 and YOLOv3_tiny to detect the maize crops and performed better than traditional image segmentation methods. A two-stage soybean leaf segmentation model based on leaf localization and guided segmentation achieved high accuracy (AP=0.976, AR=0.981), effectively handling overlapping leaves (Wang et al., 2023). YOLOv8-seg has been enhanced with Ghost and BiFPN modules, reaching a Dice score of 86.4% on the CVPPP Leaf Segmentation Challenge, especially improving small leaf segmentation (Wang et al., 2024b). For tobacco, improvements to MASK RCNN with feature fusion and hybrid attention achieved Avg.MIoU of 85.10% and Avg.MPA of 84.94%, and the Segment Anything Model (SAM) demonstrated robust segmentation across growth stages (Zhang et al., 2023). These studies highlight the effectiveness of deep learning for high-precision leaf segmentation under complex conditions. However, these studies are all based on RGB images, and research on leaf segmentation using multispectral grayscale images remains limited. Therefore, this study proposes an instance segmentation algorithm specifically for multispectral images, aiming to accurately separate individual leaves and improve the estimation accuracy of leaf-related traits.

Uneven the total nitrogen and nicotine content vertical distribution also pose a great challenge for accurate estimation in field. As contents vary substantially across different leaf positions, single-type feature fails to achieve satisfactory performance. In recent years, the integration of spectral and texture features has emerged as a promising approach for crop quality prediction, driven by advancements in deep learning and data analytics. For instance, when using the canopy spectral and texture features obtained by drones to estimate the leaf area index (LAI) of plants, integrating spectral and texture information significantly improves the prediction accuracy of the model, outperforming the model that only uses spectral features (Yuan et al., 2023; Qiao et al., 2024). Moreover, the fusion method of spectral and texture features has also been widely applied in the research of estimating plant chemical components. Relevant studies combined continuous wavelet transform (CWT) to extract canopy spectral and texture information of winter wheat, and fused thermal infrared temperature features, constructing a CNN and LSTM deep learning model to estimate the leaf water content (LWC) of winter wheat. The results showed that the multi-source feature fusion significantly improved the prediction accuracy (Yang et al., 2025). Similarly, in the tobacco field, there are also studies that use color, shape and texture features to train convolutional neural networks for monitoring the moisture content of cigar leaves (Hao et al., 2023). Therefore, we want to explore if deep learning-based fusion of spectral and texture features can improve the estimation of the chemical components of tobacco at different vertical positions.

To address the problem of leaf missegmentation in complex environment and the low accuracy arising from the uneven vertical distribution of chemical components at canopy scale, this study aims to propose an improved leave segmentation method and integrate spectral and texture data based on deep learning for estimation accuracy enhancement of total nitrogen and nicotine content. The specific objectives are as follows:

- To propose an instance segmentation on tobacco leaves based on the improved YOLOv8 model compare it with other commonly used methods, and validate its performance at both leaf and canopy scales.
- To extract spectral and texture features based on GLCM, Local Binary Pattern, Fourier Transform, Gabor Filter, and Wavelet Transform, analyze the correlations between features and total nitrogen and nicotine content, and employ feature screening methods to identify sensitive feature combinations.
- To establish estimation models based on fusion of spectral and texture features by different deep learning methods, compare their performance at leaf and canopy scales, and conduct visualization of spatial distribution of total nitrogen and nicotine in tobacco plants in field.

## 2 Materials and methods

### 2.1 Experimental design

In this study, the growth gradient of tobacco plant was controlled by fertilizer gradient experiment. The experimental variety was Zhongyan 100, and the experiment site was Changqiao Town, Pingdingshan City, Henan Province. Five fertilizer gradient treatments were designed (T0, T50, T100, T150, and T200), representing nitrogen application rates of 0%, 50%, 100%, 150%, and 200% of the local standard nitrogen application rate. Each treatment was implemented in three independent plots (biological replicates), yielding 15 plots in total. To prevent the influence of different fertilizer gradients, an isolation row was placed between treatments. Additionally, each fertilizer gradient was managed according to standardized fertilization methods to ensure the representativeness and scientific validity of the experimental results.

### 2.2 Data acquisition

#### 2.2.1 Multispectral image acquisition

The MicaSense Altum-PT multispectral camera was used for image capture. The camera has a total of 6 multispectral lenses, and a single capture can obtain images with six different spectral bands, as shown in **Table 1**.

**Table 1:** Band information of MicaSense Altum-PT multispectral camera.

Band name	Center wavelength	Bandwidth	Resolution
Blue	475nm	32nm	2064 × 1544
Green	560nm	27nm	2064 × 1544
Red	668nm	16nm	2064 × 1544
Red Edge	717nm	12nm	2064 × 1544
Near Infrared	842nm	57nm	2064 × 1544
Pancolour	634.5nm	463nm	4112 × 3008

**Figure 1:**
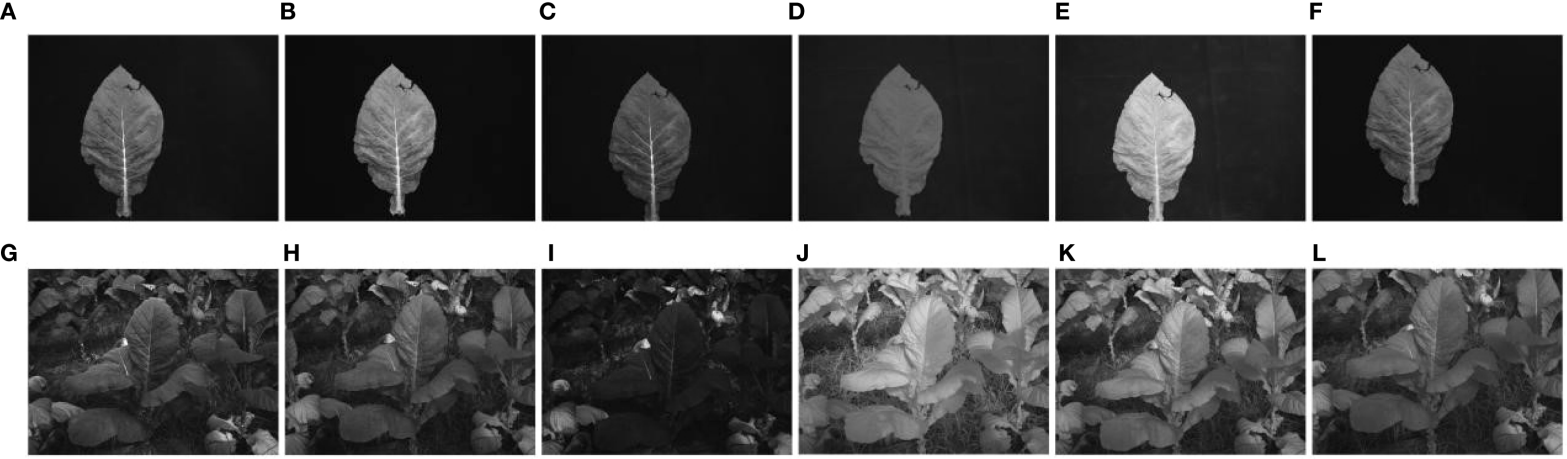
Multispectral images at leaf and canopy scales. **(A–F)** and **(G–L)** are tobacco leaf pictures in blue, green, red, red-edged, near-infrared and panchromatic bands, respectively.

A total of 13 complete tobacco plants were selected from the five treatments in the experimental field (T0, T50, T100, T150, and T200), with 3 plants selected from T0, T50, and T100, and 2 plants selected from T150 and T200. Each plant was photographed using a multispectral camera from three different angles, resulting in a total of 234 (13 × 3 × 6) multispectral images of whole plants. Subsequently, the selected plants were brought back to the laboratory, where individual leaves from each plant were photographed using the same camera for sampling. In total, 122 leaves were collected from the 13 plants. After excluding 2 leaves with significant damage or small areas, 120 leaves were used in the experiment, resulting in 720 (120 × 6) single-band multispectral images. **Figure 1** shows the spectral images of individual leaves and whole plants in different spectral bands.

#### 2.2.2 Determination of chemical constituents in tobacco leaves

Fresh tobacco leaf samples were freeze-dried using a freeze dryer (FreeZone2.5Plus, LABCONCO, USA). After freeze-drying, the tobacco leaves were analyzed for nicotine, and total nitrogen content (mass fraction) following the methods outlined in standards YC/T 468–2013 and YC/T 161-200.

### 2.3 Data processing

#### 2.3.1 Tobacco leaf segmentation model

To accurately obtain the image region data of each tobacco leaf and eliminate the influence of the background, this study performs instance segmentation on tobacco leaves based on the improved YOLOv8 model (AO-YOLOv8) to remove the background. AO-YOLOv8 enhances the feature learning ability of the model and improves the accuracy of leaf segmentation by incorporating the Aggregated Attention mechanism (Shi, 2023) and the Online Reparameterized Convolutional Module (Hu et al., 2022) into YOLOv8.

**(1)** C2f_AA

**Figure 2:**
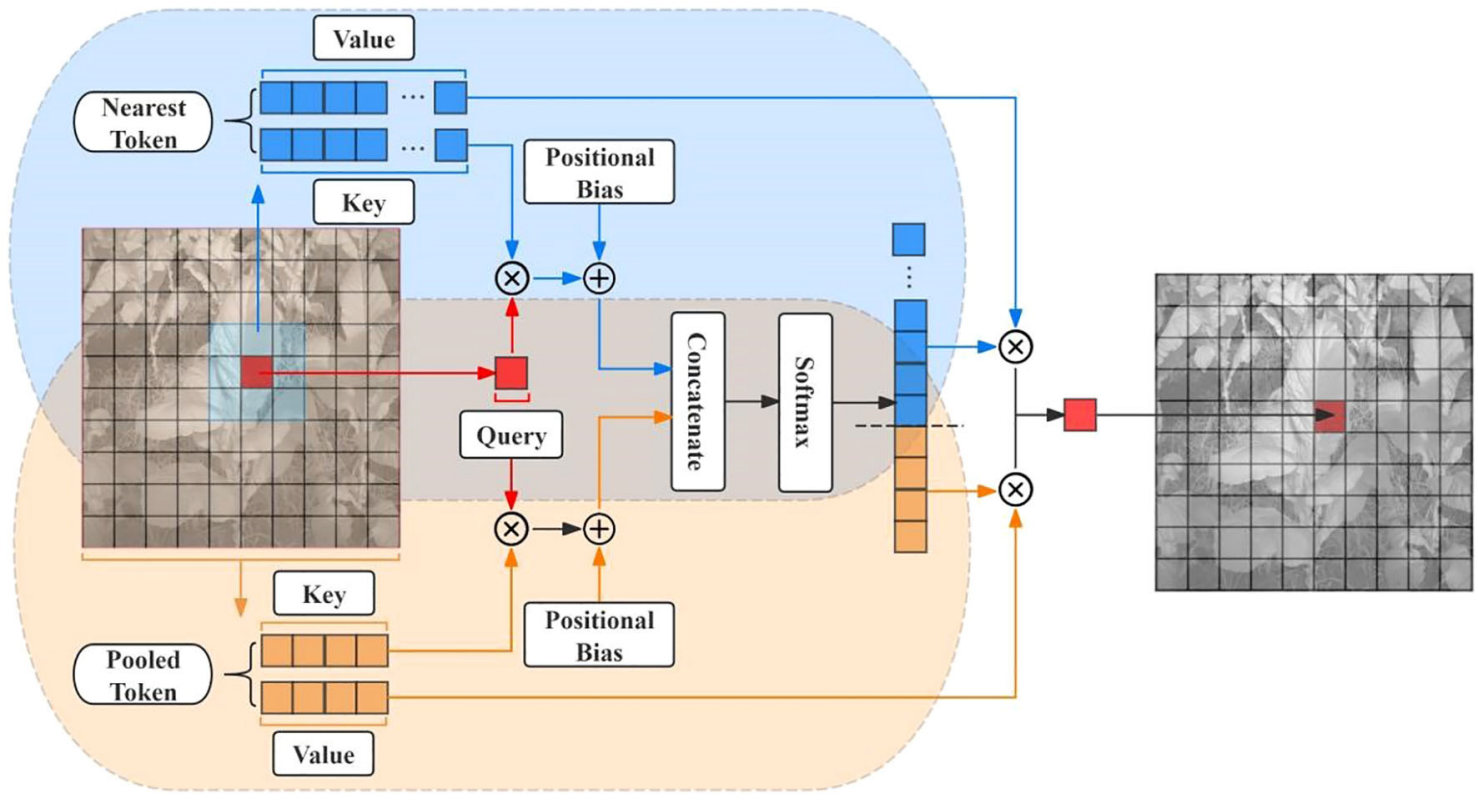
Workflow flowchart for aggregated attention.

In the canopy scale of tobacco plants, large leaves may overlap with other leaves or soil in the background. Since multispectral images are presented in grayscale, the color differences between leaves and between leaves and the soil are minimal, which can lead the model to mistakenly recognize overlapping leaves as the same leaf. To address this issue, this study proposes the C2f_AA module to replace the C2f module in the backbone of YOLOv8. In this study, the module adds an Aggregated Attention on the basis of the original C2f. The Aggregated Attention mechanism mimics the biological visual ability to dynamically adjust focus and employs a dual-path design that combines fine-grained local perception with coarse-grained global perception. This dual-path design has been validated to capture information at different levels, improving the segmentation accuracy of small regions while ensuring overall segmentation integrity (Shi, 2023). Its workflow is shown in **Figure 2**.

The first path focuses on fine-grained features around each target pixel. Through detailed local perception, the model can better handle overlapping areas between tobacco leaves, the edges of the leaves, and cluttered background information. By extracting features from local areas, the first path can focus on the details of overlapping and blurred leaf boundaries, ensuring that the model accurately segments individual tobacco leaves and avoids misidentifying overlapping leaves as a single one. This improves the segmentation accuracy of overlapping regions.

The second path performs pooling on the entire feature map to obtain coarse-grained global information. The role of this path is to focus on the contextual information of the entire image, helping the model understand the overall structure of the background and the tobacco leaves. Through global perception, the second path ensures the completeness of the tobacco leaf segmentation. In multispectral images, the background interference can be very similar to the color of the tobacco leaves, leading to missegmentation. Global perception, by capturing large-scale features in the image, effectively distinguishes the background from the tobacco leaf region and ensures that the segmentation result is not influenced by the cluttered background.

**(2)** OREPAGELAN

**Figure 3:**
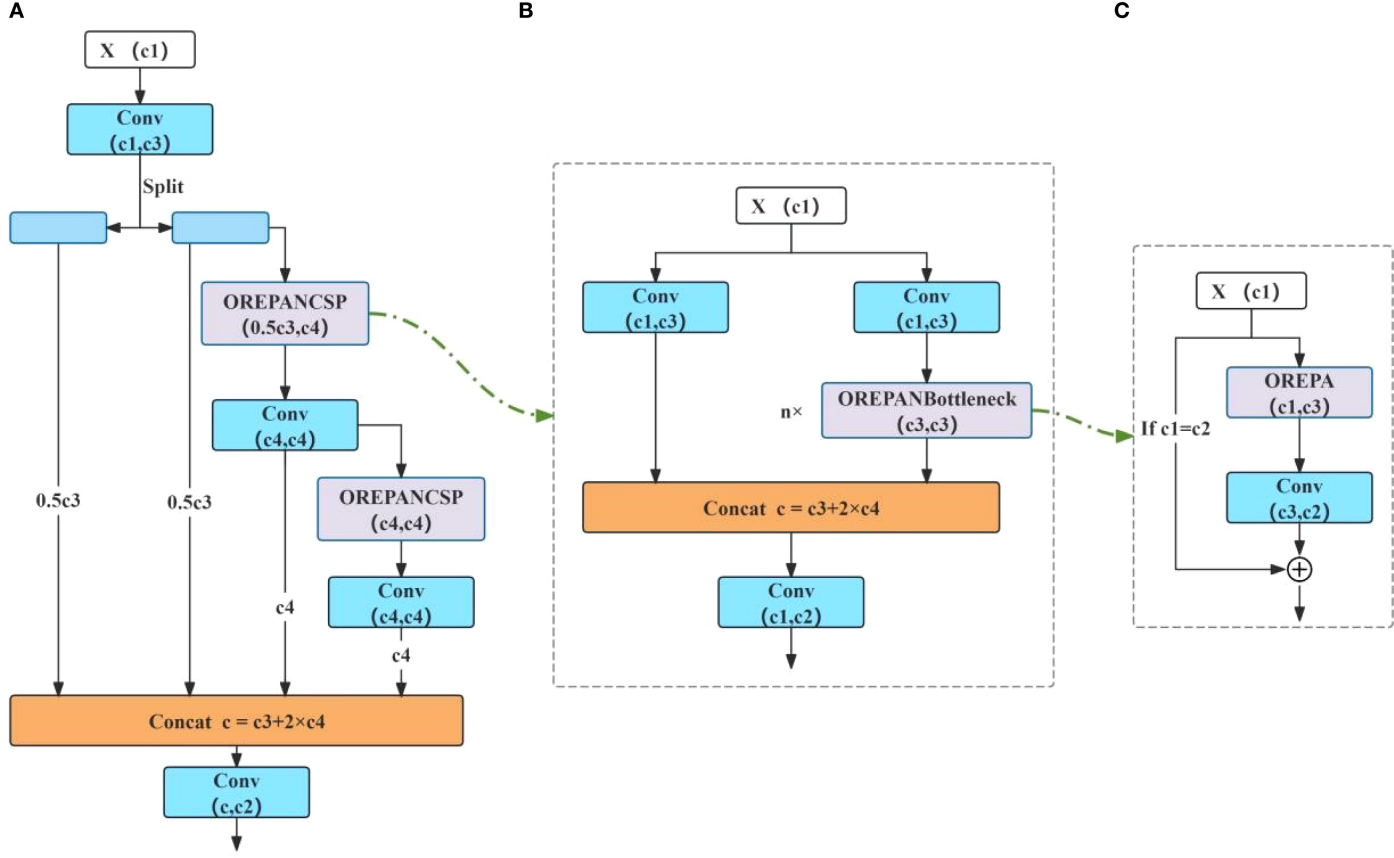
Modified module OREPAGELAN.

In addition, the GELAN (Wang et al., 2025) module, which integrates CSPNet (Wang et al., 2020) and ELAN (Wang et al., 2022), was used to replace the C2f module in the neck of YOLOv8. And then, the re-parameterized convolutional module OREPA was used to improve GELAN, naming the modified module OREPAGELAN. The integration of OREPA and GELAN enables multi-branch feature extraction during training and re-parameterization of the branches into a single convolutional block during inference. This design allows the model to learn diverse features across multiple scales and viewpoints while maintaining computational efficiency (Hu et al., 2022). Therefore, OREPAGELAN enhances the network’s expressive power without introducing significant complexity. The overall structure is shown in **Figure 3**. OREPA is a two-stage multi-branch reparameterized convolution module. The first stage, called the linearization stage, simplifies complex computational blocks by removing the nonlinear normalization layers and introducing a special linear scaling layer. The second stage, known as the block compression stage, compresses the already linearized blocks from the first stage and simplifies them into a single convolutional layer.

**Figure 4:**
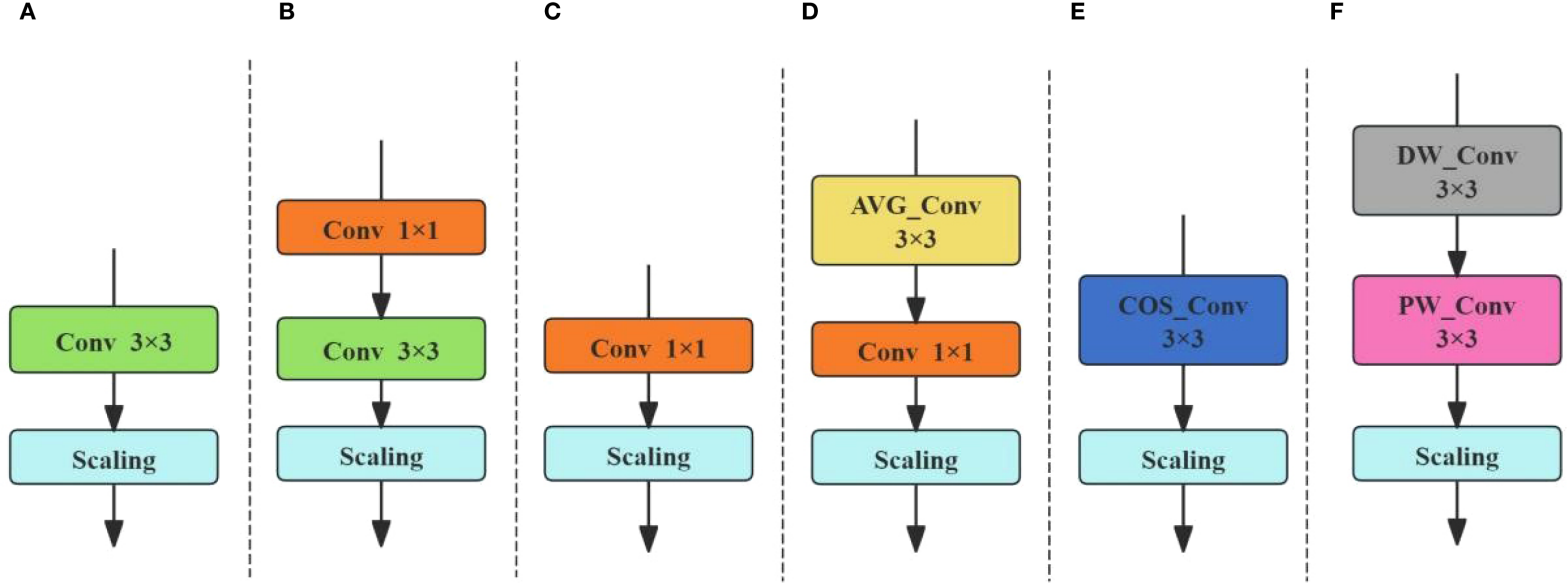
Six branches of OREPA. **(A)** 3×3 convolution block. **(B)** serial convolution block. **(C)** 1×1 serial convolution block. **(D)** mean convolution block. **(E)** cosine convolution block. **(F)** linear depth-separable convolution block.

In this study, OREPA consists of six branches, each of which learns different features through multiple branches, as shown in **Figure 4**. These branches are: 3×3 convolution block, serial convolution block, 1×1 serial convolution block, average convolution block, cosine convolution block, and linear depthwise separable convolution block. By introducing the OREPA reparameterization module, the model can learn diverse features of the image from multiple branches at different angles and scales. This enables the model to learn features at different levels and types, providing a more comprehensive understanding of both the details and global information of the tobacco leaf. Additionally, by using linearization and block compression, the features extracted by each branch are fused, improving model accuracy while reducing model complexity.

This study used commonly used evaluation metrics mAP50 and mAP50–95 in instance segmentation to evaluate the segmentation performance of the AO-YOLOv8 model. mAP50 represents the average accuracy calculated at a threshold of IoU (Intersection over Union)=0.5, while mAP50–95 calculates the average accuracy at multiple thresholds in steps of 0.05 between IoU=0.5 and IoU=0.95, and takes the mean. Meanwhile, this article introduced the semantic segmentation domain evaluation index mIoU to assess the degree of overlap between the predicted mask and the true mask for each leaf. Additionally, to validate the capability of our proposed segmentation method, comparative experiments were conducted including the AO-YOLOv8 model and other YOLO-series models (e.g., YOLOv5, YOLOv7, and YOLOv9), along with classical instance segmentation models such as Mask R-CNN and YOLACT.

#### 2.3.2 Feature extraction

**(1)** Spectral feature extraction

**Figure 5:**
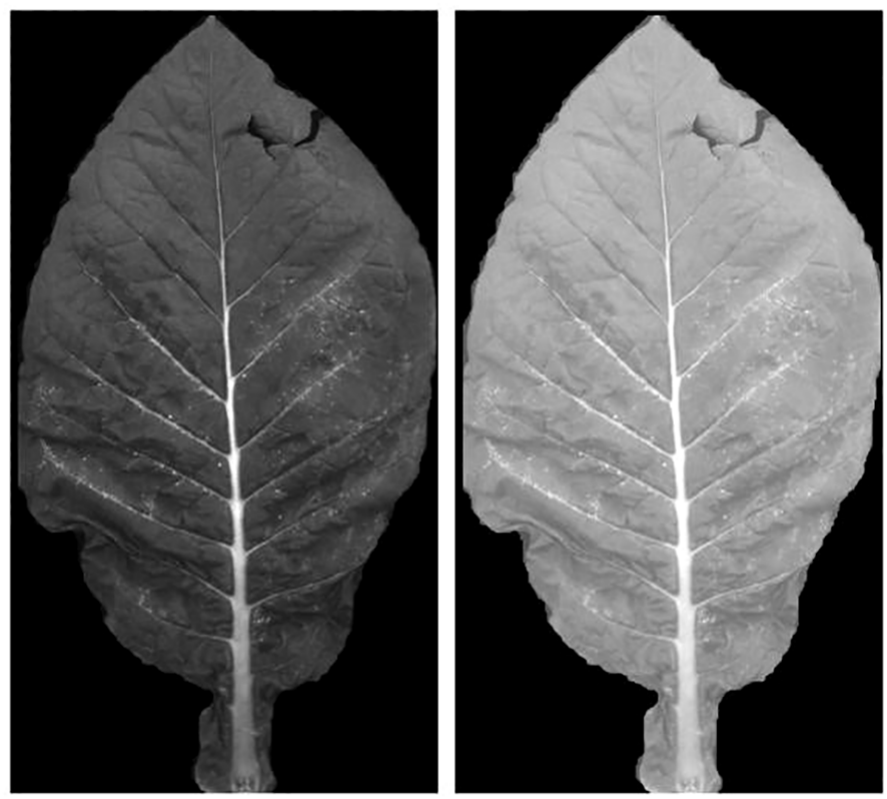
Original image (left) and reflectance-corrected image (right).

The images after segmentation were calibrated using ENVI software in combination with a calibration board, and the difference before and after calibration is shown in **Figure 5**. After calibration, the images were used to extract the average spectral features of the leaves. During the extraction process, pixels with black backgrounds (R = G = B=0) were ignored. Spectral features from six bands—blue, green, red, red-edge, near-infrared, and panchromatic—were extracted for each tobacco leaf.

**(2)** Texture feature extraction

Texture features reflect the physical structure, micro-distribution, and indirectly the chemical properties of materials. In this study, five texture analysis methods were selected: Gray-Level Co-occurrence Matrix (GLCM), Local Binary Pattern (LBP), Fourier Transform, Gabor Filter, and Wavelet Transform. They provide complementary descriptions of leaf texture at different scales and domains. Specifically, GLCM was used to extract contrast, dissimilarity, homogeneity, energy, and correlation. For LBP, a neighborhood radius of 1 with 8 surrounding points was applied, and the distribution of the resulting local patterns was calculated to generate 10 statistical features. Fourier Transform was employed to obtain the amplitude and phase spectrums, from which the mean and standard deviation of both magnitude and phase were derived. Gabor filters were applied with four scales and four orientations, extracting mean, standard deviation, and energy for each combination. Wavelet Transform decomposed the image into four sub-bands (LL, LH, HL, and HH), and energy, variance, and entropy were calculated for each sub-band.

Each tobacco leaf corresponds to six spectral images, five texture analysis methods were applied to each spectral image, resulting in a total of 474 texture features. These five methods were chosen because they capture complementary aspects of leaf texture: GLCM characterizes statistical gray-level relationships, LBP encodes local venation and microstructural details, Fourier describes global periodic patterns, Gabor emphasizes orientation and scale-specific traits, and Wavelet provides multi-scale representations. As nitrogen and nicotine contents are closely related to venation and surface texture, this combination offers a more comprehensive description than any single method alone.

#### 2.3.3 Feature screening method

The initial extraction yielded 480 features (474 texture + 6 spectral), feature screening was necessary to reduce computational redundancy and highlight the most informative descriptors for nitrogen and nicotine prediction. Four representative methods were employed: (i) Pearson correlation threshold (0.3, 0.5, 0.6) to retain features with higher correlation to nitrogen or nicotine, (ii) variance threshold to exclude features with little variation, (iii) LASSO, which uses L1 regularization to shrink irrelevant feature weights to zero, and (iv) Elastic Net, which combines L1 and L2 regularization to robustly select among correlated features. The resulting subsets (**Tables 2**, **3**) ensured that subsequent deep learning models could focus on the most relevant features, improving efficiency and generalization.

**Table 2:** Number of retained features for each screening method (laboratory leaves).

Dataset	Characteristic quantity (Total Nitrogen)	Characteristic quantity (Nicotine)
Spectrum	6	6
Spectrum + Texture	480	480
Spectrum + Texture(Pearson>0.3)	149	158
Spectrum + Texture(Pearson>0.5)	56	87
Spectrum + Texture(Pearson>0.6)	5	–
Spectrum + Texture (Variance Threshold)	352	352
Spectrum + Texture(LASSO)	35	42
Spectrum + Texture(Elastic Net)	64	83

**Table 3:** Retention characteristics of each screening method (leaves of tobacco plants in the field).

Dataset	Characteristic quantity (Total Nitrogen)	Characteristic quantity (Nicotine)
Spectrum	6	6
Spectrum + Texture	480	480
Spectrum + Texture(Variance threshold)	372	372
Spectrum + Texture(LASSO)	41	44
Spectrum + Texture(Elastic Net)	68	80

#### 2.3.4 Data augmentation

This study employed three data augmentation methods: Gaussian noise addition, random scaling, and random offset. Gaussian noise was added to the data to make its values follow a normal distribution, with a mean of 0 and a variance of 0.01, simulating potential random errors or interference during data acquisition. Random scaling was applied by multiplying the data by a randomly selected factor within the range of (0.9, 1, 1), adjusting the overall magnitude of the data. Random offset involved adding a random value to the entire dataset to simulate signal baseline drift or systematic measurement errors. This method can effectively increase or decrease all data points by a fixed value, with the random offset range set to (-0.01, 0.01) in this study.

#### 2.3.5 Dataset partitioning and estimation model establishment

In this study, the dataset was divided into training, validation, and test sets in a 7:2:1 ratio. To prevent any influence on model validation during training and testing after training, data augmentation using Gaussian noise addition, random scaling, and random offset was applied only to the training set.

In this experiment, four deep neural network models, namely Long Short-Term Memory Network (LSTM), Recurrent Neural Network (RNN), multi-layer Perceptron (MLP), and Fully Connected Neural Network (FCNN), were adopted to predict the total nitrogen and nicotine content of tobacco leaves. (i)RNN aims to utilize its sequence processing capability to mine the spatial ordered correlation of texture features; (ii)LSTM overcomes the memory limitations of RNN in long sequences through its unique gate system and better captures complex texture relationships; (iii)MLP is used as a simple and universal benchmark model to handle fixed-dimensional inputs; (iv)FCNN focuses on high-dimensional feature fusion and end-to-end prediction, effectively combining multi-source information such as spectra and textures.

The performance of the estimation models was evaluated with determination coefficient of test set (R^2^) and the root mean square error (RMSE).

## 3 Results

### 3.1 Correlation analysis of total nitrogen and nicotine with spectral and texture features

**Figure 6:**
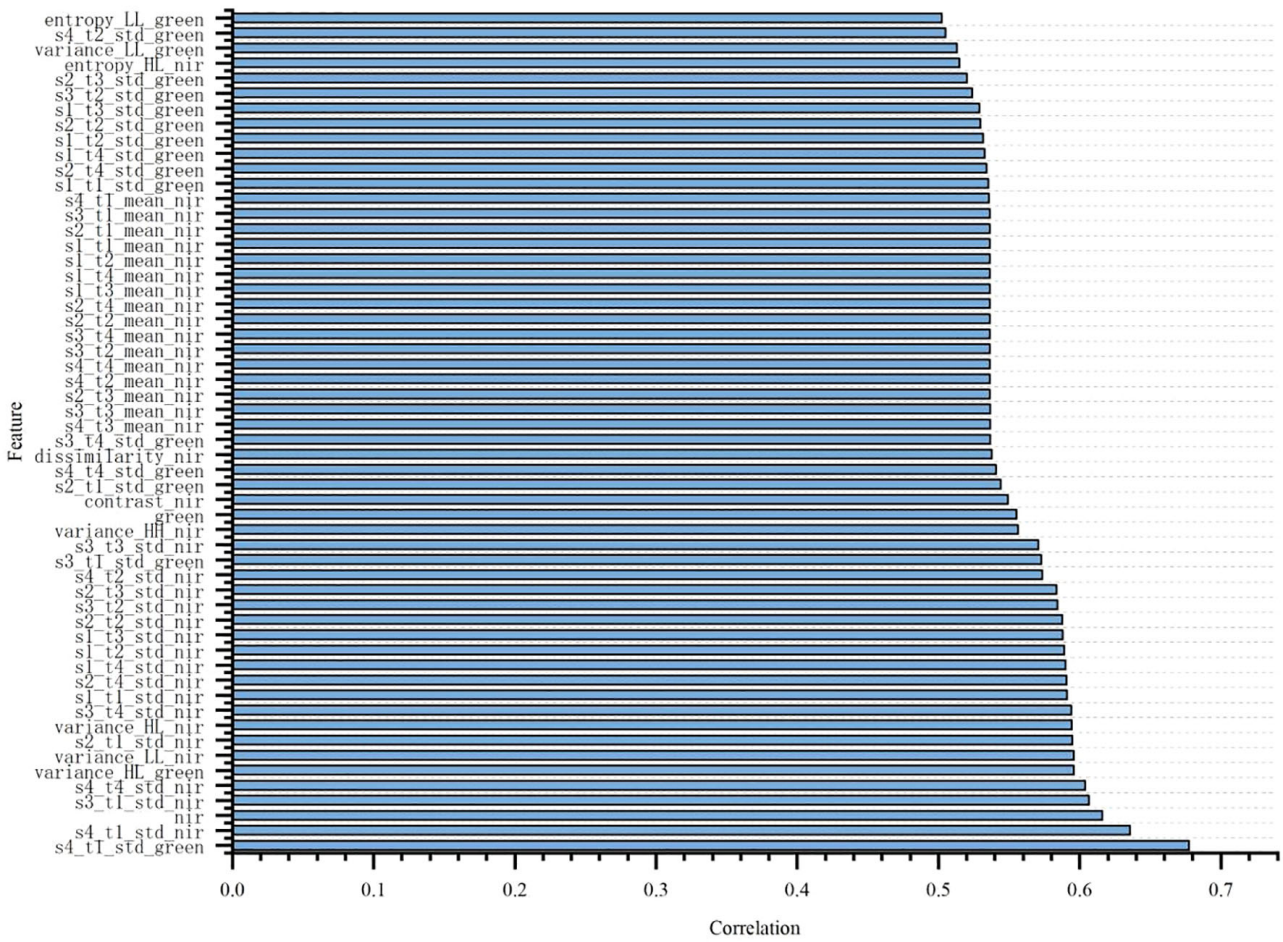
Correlation between spectral and texture features and total nitrogen.

**Figure 7:**
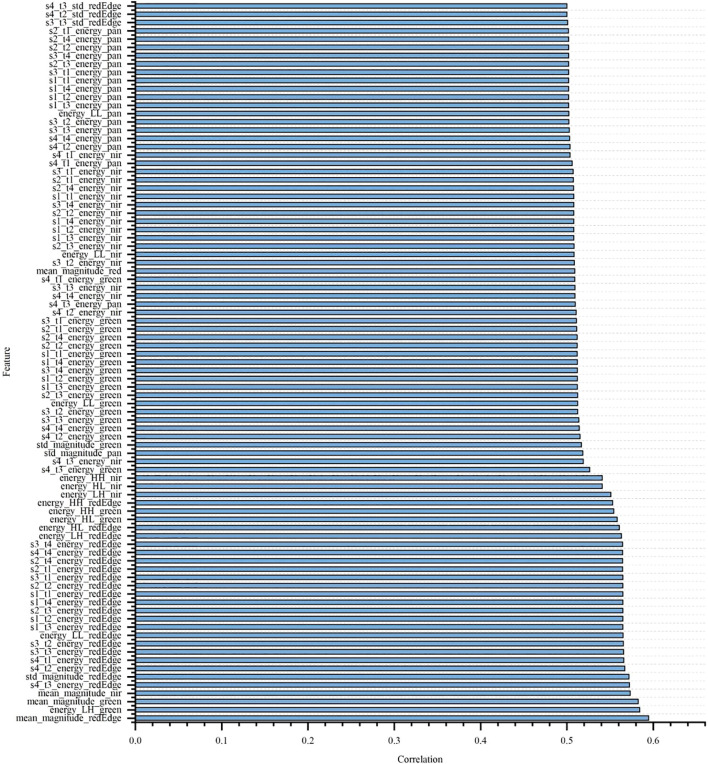
Correlation between spectral and texture features and nicotine.

To explore the correlation between different spectral features and texture features with nitrogen and nicotine content, the Pearson correlation coefficient method was used to analyze the laboratory single tobacco leaves, as shown in **Figures 6** and **7**. Due to the large number of features, only those with Pearson correlation coefficients greater than 0.5 were presented. From the figures, it was evident that most texture features showed a significant correlation with total nitrogen and nicotine content. Additionally, in terms of spectral features, the correlation coefficients of the NIR and green bands with total nitrogen content were 0.618 and 0.557, respectively, indicating a strong correlation. The strong correlation of the NIR and green bands with total nitrogen content is due to two factors: (i) nitrogen influences chlorophyll concentration, which strongly absorbs blue and red light while reflecting green light, making the green band sensitive to nitrogen content; (ii) nitrogen affects leaf internal structure and water content, which primarily alter NIR reflectance, resulting in a high correlation between NIR bands and total nitrogen (Wang et al., 2021). However, no spectral features had a correlation coefficient greater than 0.5 with nicotine content.

### 3.2 Performance analysis of proposed tobacco leaf segmentation model

#### 3.2.1 Validation of AO-YOLOv8 model

To verify the effectiveness of the improved model, three ablation experiments were conducted in this section. The C2f_AA and OREPAGELAN modules were sequentially applied to the YOLOv8 model to evaluate the contribution of each improvement to the multispectral tobacco leaf segmentation task. The results of the ablation experiments are shown in **Table 4**.

**Table 4:** Ablation experiment.

No.	Model	mAP50/%	mAP50-95/%	mIoU/%
1	YOLOv8	81.6	57.4	76.6
2	YOLOv8 + C2f_AA	86.0	62.0	82.0
3	YOLOv8 + C2f_AA + OREPAGELAN	**87.3**	**67.3**	**83.4**

Bold values indicate the best performance under each evaluation metric.

YOLOv8 achieved 81.6%, 57.4% and 76.6% in mAP50, MAP50–95 and mIoU, respectively. On this basis, after the introduction of C2f_AA, the model had a significant improvement in all evaluation indexes, mAP50 increased to 86.0%, MAP50–95 increased to 62.0%, mIoU increased to 82.0%, indicating that the improvement enhances the model’s ability to detect and segment target instances. After the further introduction of OREPAGELAN, the model performance was further optimized, among which mAP50 increased to 87.3%, MAP50–95 increased to 67.3%, mIoU increased to 83.4%, and the three indexes increased by 6.99%, 17.25% and 8.88% respectively. These results validated the effectiveness of the proposed method in the case segmentation task.

**Figure 8:**
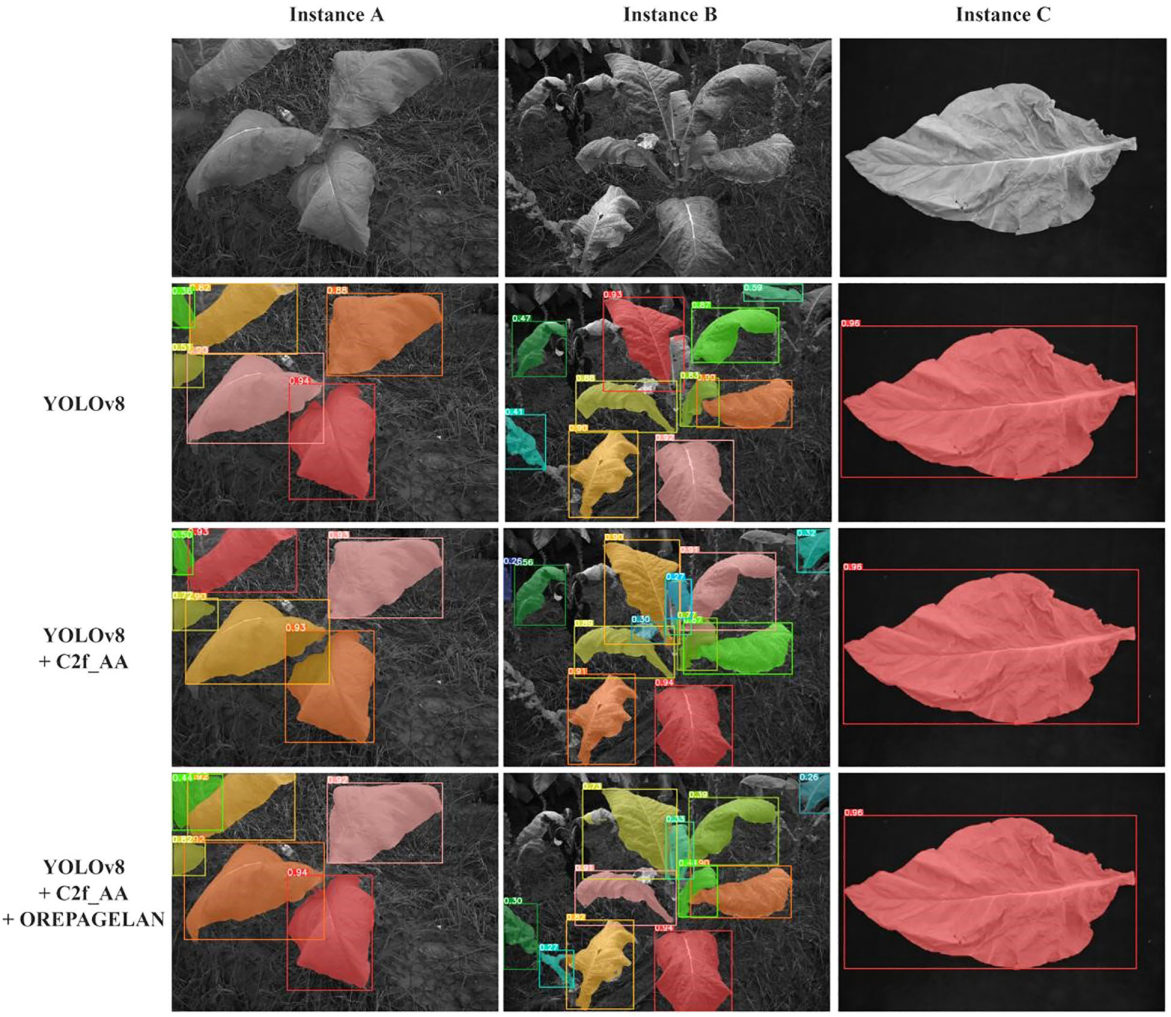
Results of ablation experiment segmentation.

**Figure 8** visually illustrates the effects of each improvement on multispectral tobacco leaf instance segmentation taken three scenes as examples. When segmenting instances A and B, the YOLOv8 model showed a tendency to miss small targets. After introducing the C2f_AA module, the model’s capability for detecting and segmenting small targets improved, with the improvement being more evident in instance B. However, overlapping segmentation masks between leaves still occurred. With the further integration of the OREPAGELAN module, the segmentation results for both instances A and B became more refined, enabling accurate detection and segmentation of small leaves while maintaining segmentation integrity and eliminating mask overlaps. For the segmentation of a single tobacco leaf in a controlled laboratory setting (Instance C), all three models achieved complete leaf segmentation.

#### 3.2.2 Comparison of mainstream case segmentation models

Furthermore, this study conducted comparative experiments between AO-YOLOv8 and other YOLO-series models (YOLOv5, YOLOv7, and YOLOv9), as well as classical instance segmentation models, including Mask R-CNN and YOLACT. All experiments were performed under the same experimental conditions. The results of each model are presented in **Table 5**. As shown in the results, AO-YOLOv8 outperformed all other models in terms of mAP50 and mIoU, while also achieving a relatively high performance in mAP50-95. These findings indicated that the proposed method can more effectively perform instance segmentation and demonstrated superior generalization ability and robustness compared to several mainstream approaches.

**Table 5:** Comparison of experimental results between AO-YOLOv8 and other segmentation methods.

No.	Model	mAP50/%	mAP50-95/%	mIoU/%
1	AO-YOLOv8	**87.3**	67.3	**83.4**
2	YOLOv7	85.0	66.3	78.0
3	YOLOv5	76.3	50.5	73.2
4	YOLOv9	84.5	**68.3**	77.4
5	Mask R-CNN	80.3	63.8	77.5
6	YOLACT	78.0	59.0	71.2

Bold values indicate the best performance under each evaluation metric.

**Figure 9:**
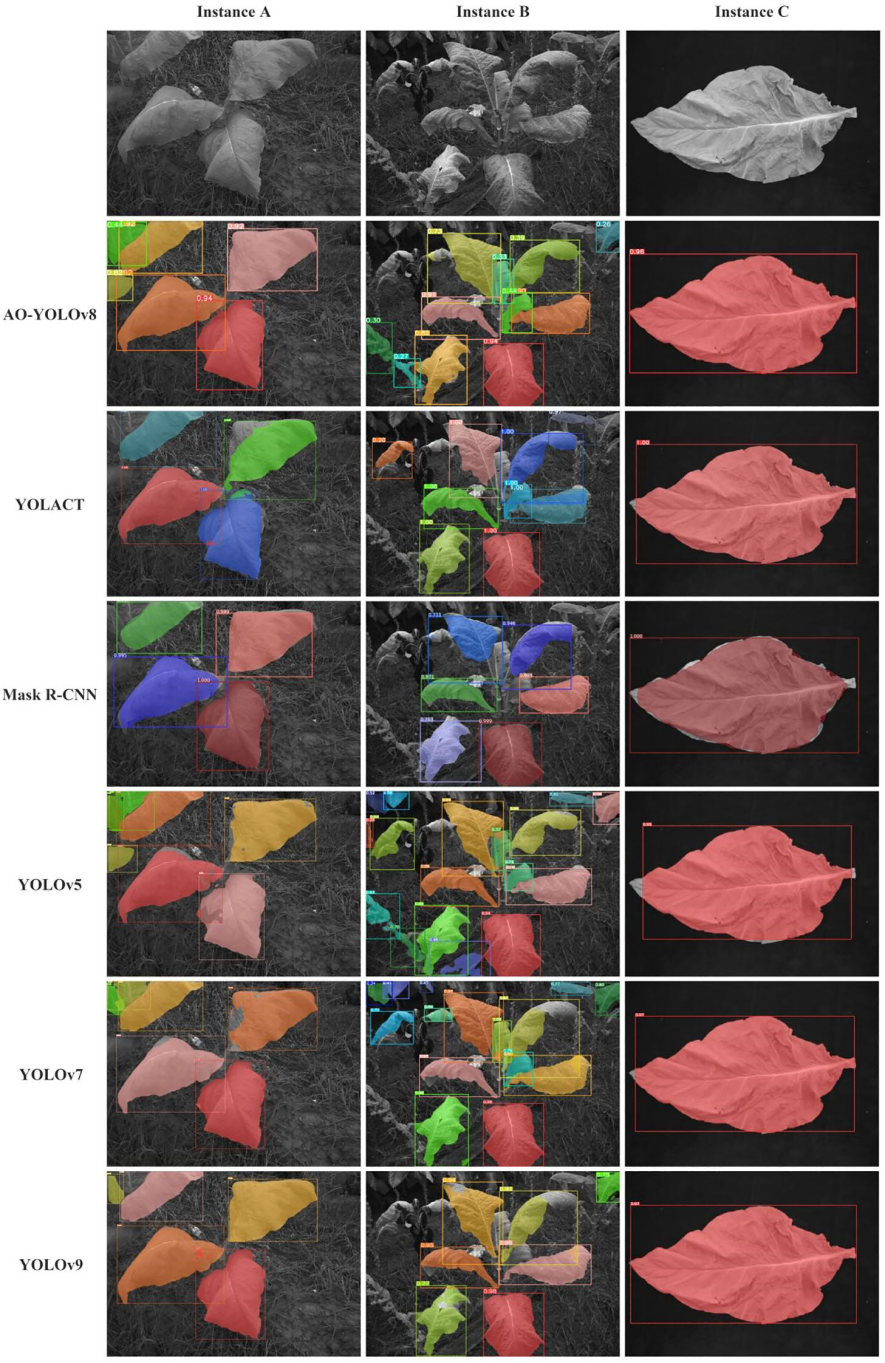
Comparison of segmentation effects of each model.

**Figure 9** illustrates the segmentation results of various models. From the comparison of segmentation performance, it can be observed that for the relatively sparse tobacco plant instance A, AO-YOLOv8 effectively segmented each leaf without mask overlapping. In contrast, other models such as YOLOv7, YOLOv5, and YOLACT exhibited incomplete leaf segmentation, while YOLOv5 and YOLOv9 suffered from mask overlap or misidentification of small leaf regions as separate leaves. Mask R-CNN achieved relatively complete segmentation but failed to identify smaller leaves. For Example B, in the case of denser blades, AO-YOLOv8 can achieve relatively accurate segmentation, while other models have problems such as incomplete recognition, mask overlap and missed targets. In the case of instance C, AO-YOLOv8 achieved complete segmentation, whereas other models showed incomplete segmentation at leaf tips, tails, and edges. These results demonstrated that the integration of C2f_AA and OREPAGELAN enhanced the model’s ability to extract tobacco leaf features in field environments, effectively distinguishing leaf boundaries and overlapping regions, thereby achieving higher segmentation accuracy.

**Figure 10:**
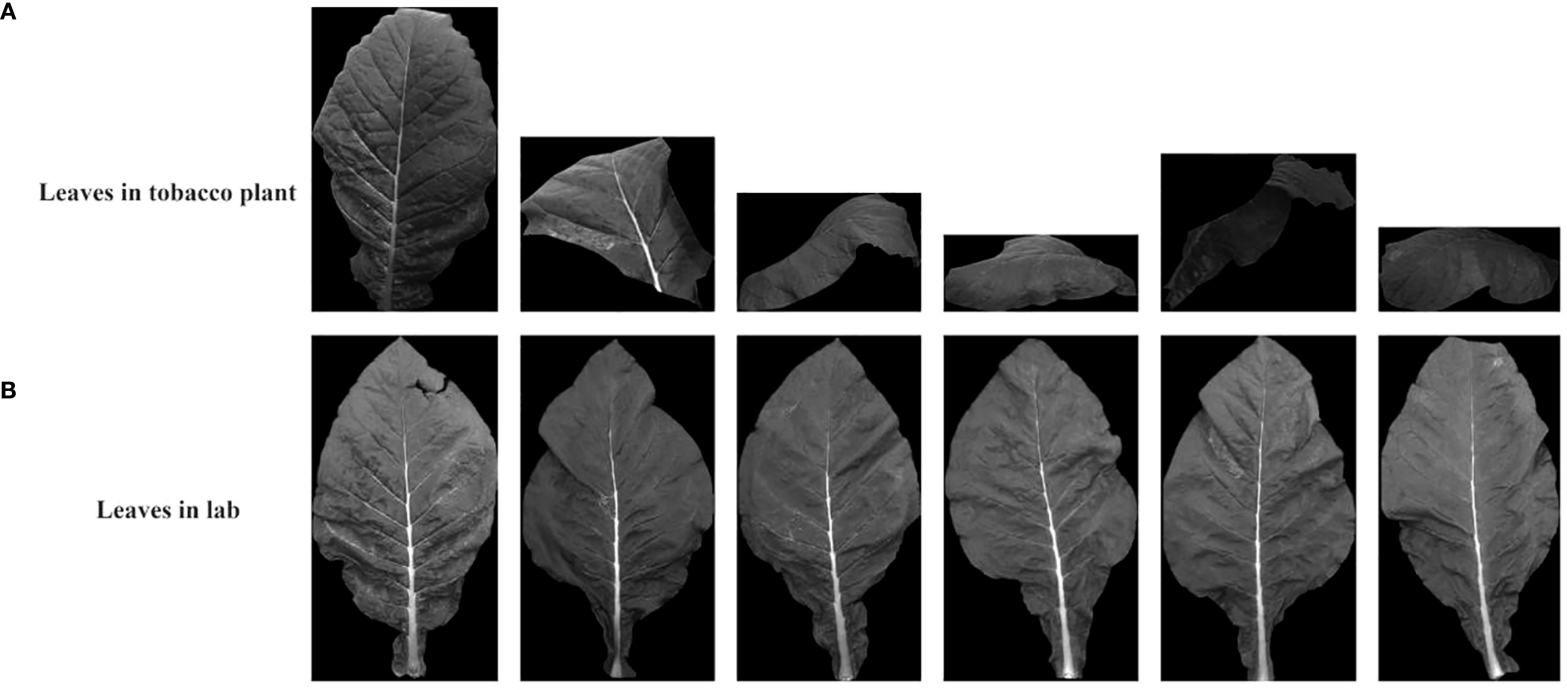
The segmentation effects of AO-YOLOv8 on tobacco leaves in the laboratory and on tobacco plants respectively.

**Figure 10** shows the segmentation results of the same tobacco leaf in different environments, and compares its segmentation effect in the field tobacco plant image and the laboratory single tobacco leaf image. Under laboratory and natural field conditions, tobacco leaves can be well segmented even if they were significantly bent or deformed.

### 3.3 Establishment of laboratory leaf scale estimation model

In this study, four deep learning models—MLP, LSTM, RNN, and FCNN—were employed to predict nitrogen and nicotine content in individual tobacco leaves captured in laboratory conditions. The experiments were conducted using the eight data types listed in **Table 2**, and the results are presented in **Table 6**.

**Table 6:** Predicted results of total nitrogen content in leaves in laboratory.

Dataset	MLP		LSTM		RNN		FCNN	
	R²	RMSE	R²	RMSE	R²	RMSE	R²	RMSE
Spectrum	0.6273	0.2480	0.6515	0.2398	0.6279	0.2419	0.6089	0.2540
Spectrum + Texture	0.7464	0.2045	**0.8634**	**0.1501**	0.6003	0.2507	0.7642	0.1972
Spectrum + Texture (Pearson>0.3)	**0.8246**	**0.1701**	0.8241	0.1704	0.6488	0.2350	**0.8550**	**0.1547**
Spectrum + Texture (Pearson>0.5)	0.7355	0.2089	0.7553	0.2010	0.5258	0.2731	0.6728	0.2324
Spectrum + Texture (Pearson>0.6)	0.6880	0.2269	0.6363	0.2450	0.6167	0.2455	0.7367	0.2084
Spectrum + Texture (Variance threshold)	0.7962	0.1834	0.7717	0.1941	0.6984	0.2178	0.7329	0.2099
Spectrum + Texture (LASSO)	0.6170	0.2514	0.5756	0.2647	0.6374	0.2388	0.7172	0.2160
Spectrum + Texture (Elastic Net)	0.7854	0.1882	0.6645	0.2353	**0.7126**	**0.2126**	0.8050	0.1794

Bold values indicate the best performance under each evaluation metric.

**Figure 11:**
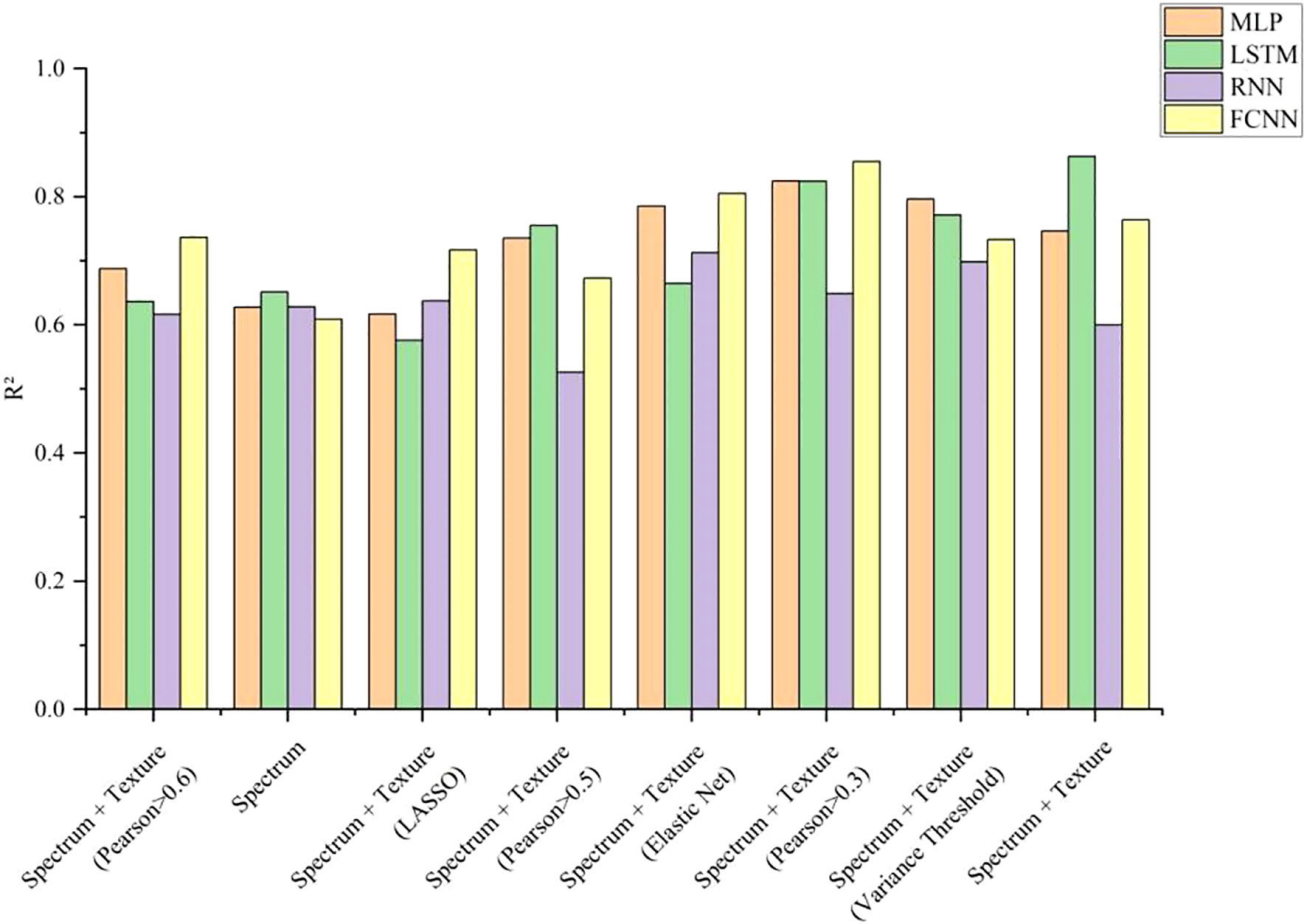
Prediction result curve of total leaf nitrogen in laboratory.

**Figure 12:**
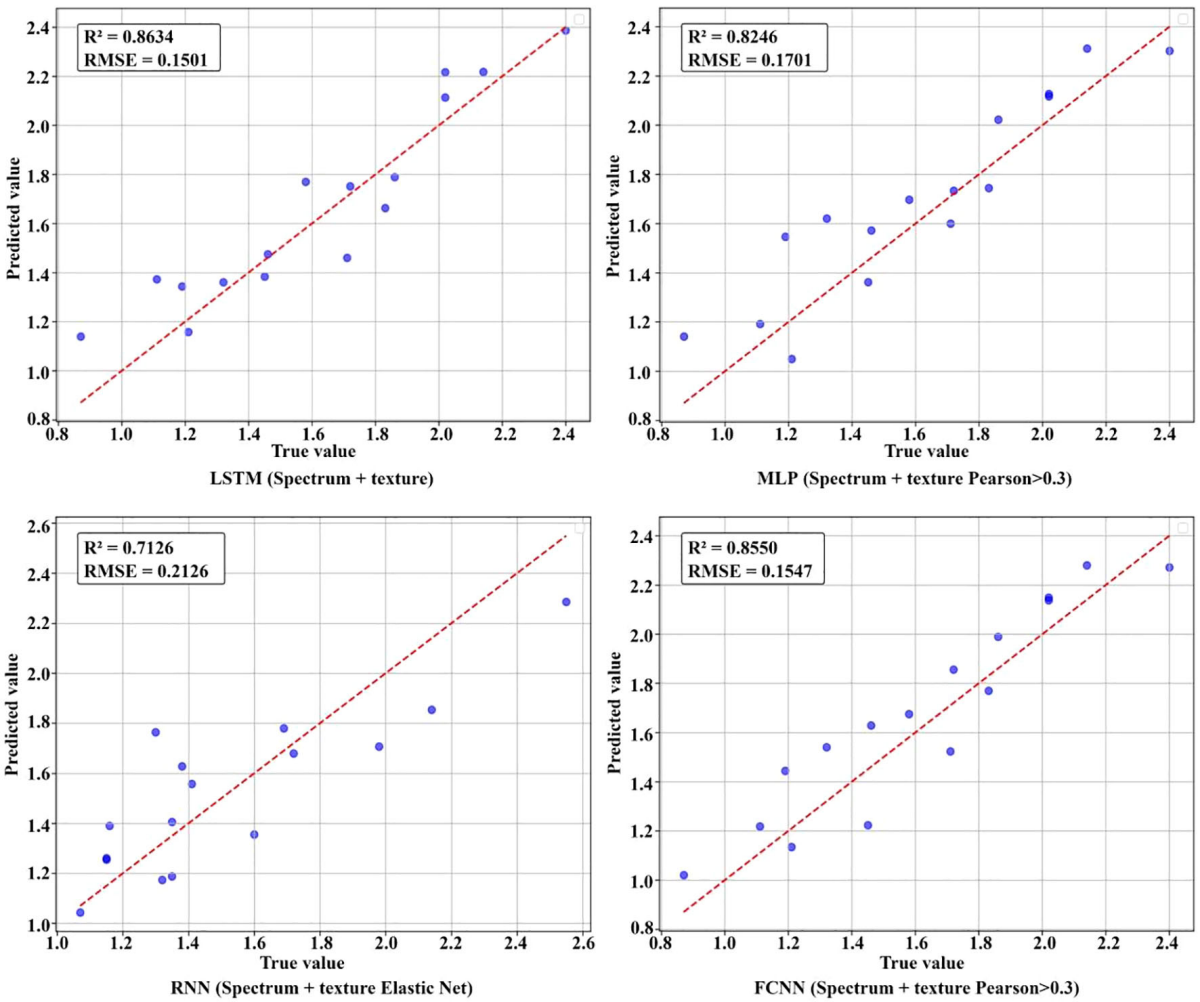
Comparison between the predicted and real values of leaf total nitrogen in the laboratory.

The experimental results indicated that when predicting total nitrogen content using only spectral data, the R² of all four deep learning models were approximately 0.62, suggesting that spectral data alone provided limited prediction accuracy. However, after incorporating texture data, the R² improved significantly. Among the models, LSTM achieved the highest R² of 0.8634 when using both spectral and texture data. The other three models also reached their highest R² when combining spectral and texture data, though the optimal feature selection method varied across models. **Figure 11** illustrates the R² for different models under various feature selection methods when predicting total nitrogen content. The results showed an increasing trend in R² as the number of features increased, indicating that the extracted texture features effectively enhanced the accuracy of total nitrogen content prediction. Deep learning models are capable of handling complex nonlinear data relationships. As network depth increases, these models can automatically learn intricate features, adapt to different data distributions and patterns, and extract multi-level features, thereby enhancing predictive accuracy for complex, multivariate data. **Figure 12** shows the fitting effect of four models on the true and predicted values in the dataset with the highest R ².

**Figure 13:**
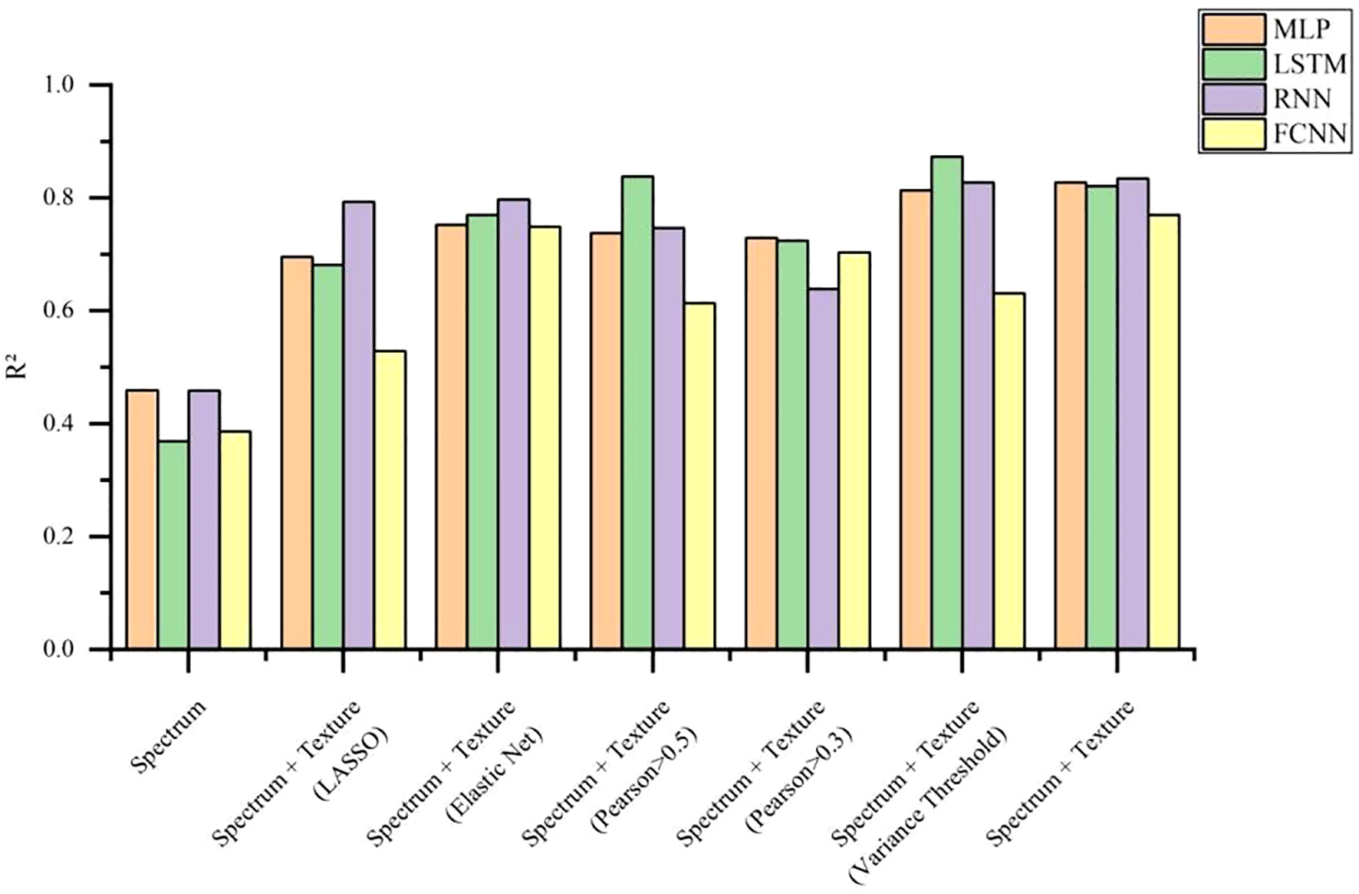
Nicotine prediction curve of leaves in laboratory.

**Figure 14:**
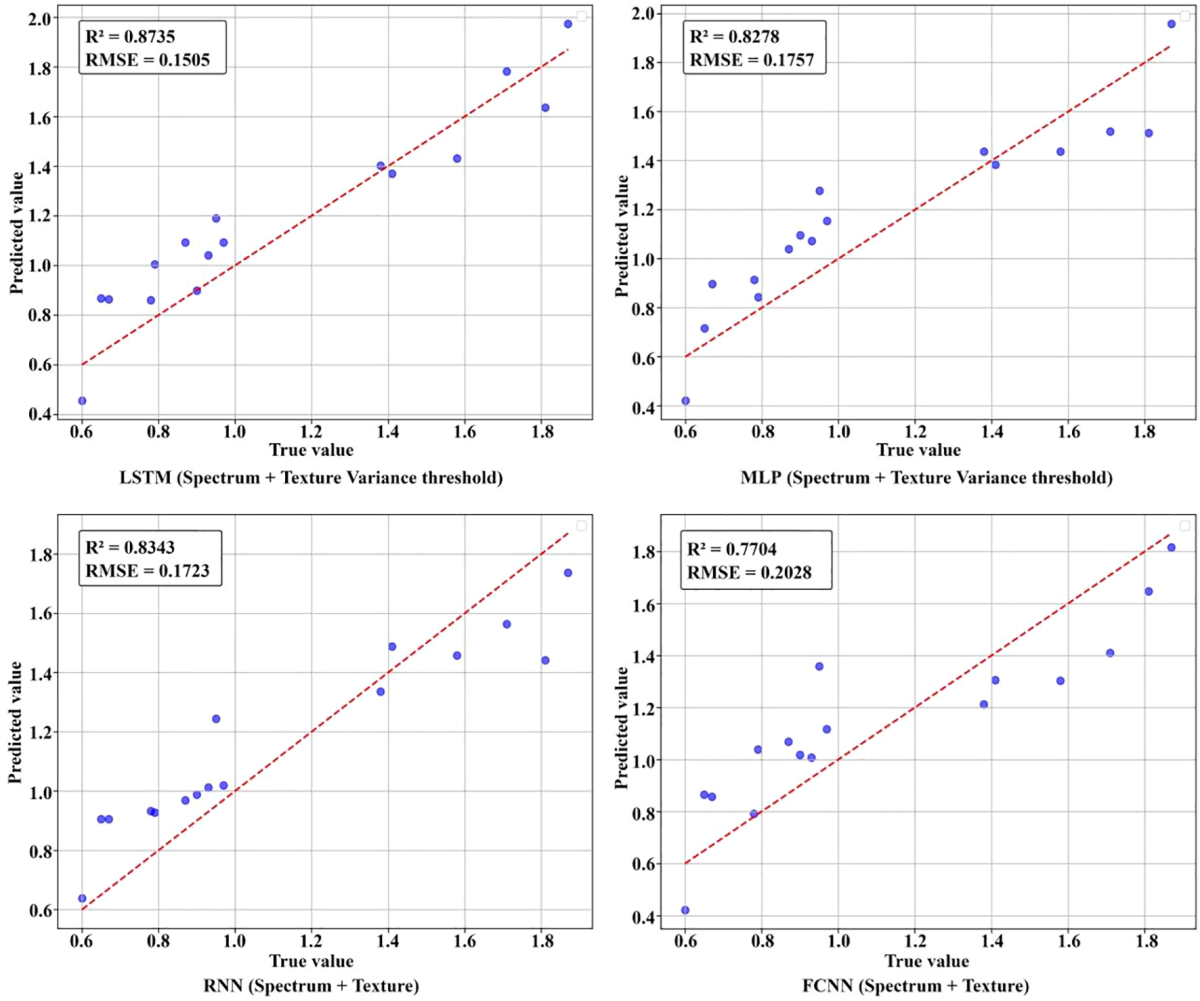
Comparison between the predicted and real values of leaf total nitrogen in the laboratory.

The results of nicotine content prediction are shown in **Table 7**, where texture features have a more significant impact on the accuracy of prediction. The MLP, RNN, and FCNN models all achieved the highest R² when using both spectral and texture data, with R² of 0.8278, 0.8343, and 0.7704, respectively. After applying variance threshold filtering to the spectral and texture data, the LSTM model achieved the highest prediction accuracy for nicotine content, with an R² of 0.8735. **Figure 13** visually illustrates the R ² of nicotine prediction under different datasets. **Figure 14** shows the fitting effect of four models on the true and predicted values in the dataset with the highest R ².

**Table 7:** Predicted results of nicotine content in leaves in laboratory.

Dataset	MLP		LSTM		RNN		FCNN	
	R²	RMSE	R²	RMSE	R²	RMSE	R²	RMSE
Spectrum	0.4594	0.3112	0.3688	0.3363	0.4587	0.3114	0.3867	0.3315
Spectrum + Texture	**0.8278**	**0.1757**	0.8211	0.1791	**0.8343**	**0.1723**	**0.7704**	**0.2028**
Spectrum + Texture (Pearson>0.3)	0.7294	0.2202	0.7247	0.2221	0.6387	0.2544	0.7034	0.2305
Spectrum + Texture (Pearson>0.5)	0.7381	0.2166	0.8378	0.1705	0.7470	0.2129	0.6138	0.2630
Spectrum + Texture (Variance threshold)	0.8139	0.1826	**0.8735**	**0.1505**	0.8275	0.1758	0.6310	0.2571
Spectrum + Texture (LASSO)	0.6958	0.2334	0.6814	0.2389	0.7929	0.1926	0.5288	0.5288
Spectrum + Texture(Elastic Net)	0.7524	0.2106	0.7699	0.2030	0.7974	0.1905	0.7492	0.2120

Bold values indicate the best performance under each evaluation metric.

### 3.4 Validation of estimation model of total nitrogen and nicotine in tobacco plants in field

**Figure 15:**
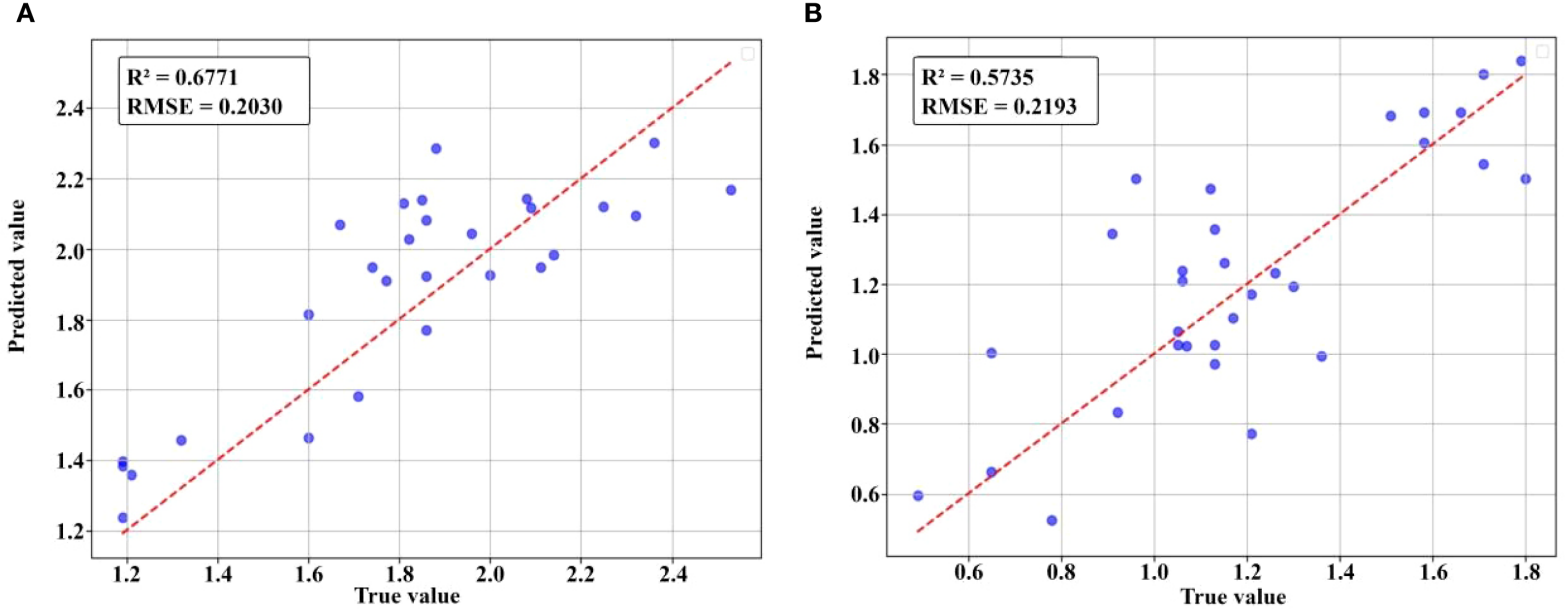
Fitting effect between predicted and true values of total nitrogen and nicotine in field tobacco plants.

The LSTM model, which achieved the highest accuracy for predicting total nitrogen and nicotine content in single tobacco leaves in the laboratory, was extended to predict total nitrogen and nicotine content at the field tobacco plant scale. This model extension required the inclusion of segmented images of field tobacco plants in the dataset for training and evaluation. A total of 195 tobacco leaves were segmented from spectral images of tobacco plants from three perspectives of 13 plants. Combined with 120 laboratory leaf samples, the dataset consisted of 315 samples, which were divided into training, validation, and test sets in a 7:2:1 ratio. The experimental results, as shown in **Table 8**, indicated that the model’s predictive performance decreased after adding segmented field tobacco leaves to the dataset compared to using only laboratory single-leaf data (**Tables 6**, **7**), which proved the difficulty of estimating the component content in field. When using only spectral data for estimation, the R² of the tobacco plant total nitrogen content prediction model was 0.3249, and the nicotine content prediction model’s R² was only 0.0667. After adding texture data, the R² values improved to 0.6771 and 0.5735, respectively. However, after applying different feature selection methods, the R² values slightly decreased, but they were still higher than those using spectral features alone. **Figure 15** shows the fitting effect of LSTM on the true and predicted values in the dataset with the highest R ².

**Table 8:** Predicted results of total nitrogen and nicotine content in tobacco plants in the field.

Dataset	Total nitrogen		Nicotine	
	R²	RMSE	R²	RMSE
Spectrum	0.3136	0.3012	0.0667	0.3043
Spectrum + Texture	**0.6771**	**0.2030**	**0.5735**	**0.2193**
Spectrum + Texture (Variance threshold)	0.6021	0.2293	0.4640	0.2458
Spectrum + Texture (LASSO)	0.5089	0.2548	0.5627	0.2329
Spectrum + Texture (Elastic Net)	0.5297	0.2493	0.5470	0.2260

Bold values indicate the best performance under each evaluation metric.

### 3.5 Visualization of spatial distribution of total nitrogen and nicotine in tobacco plants in the field

**Figure 16:**
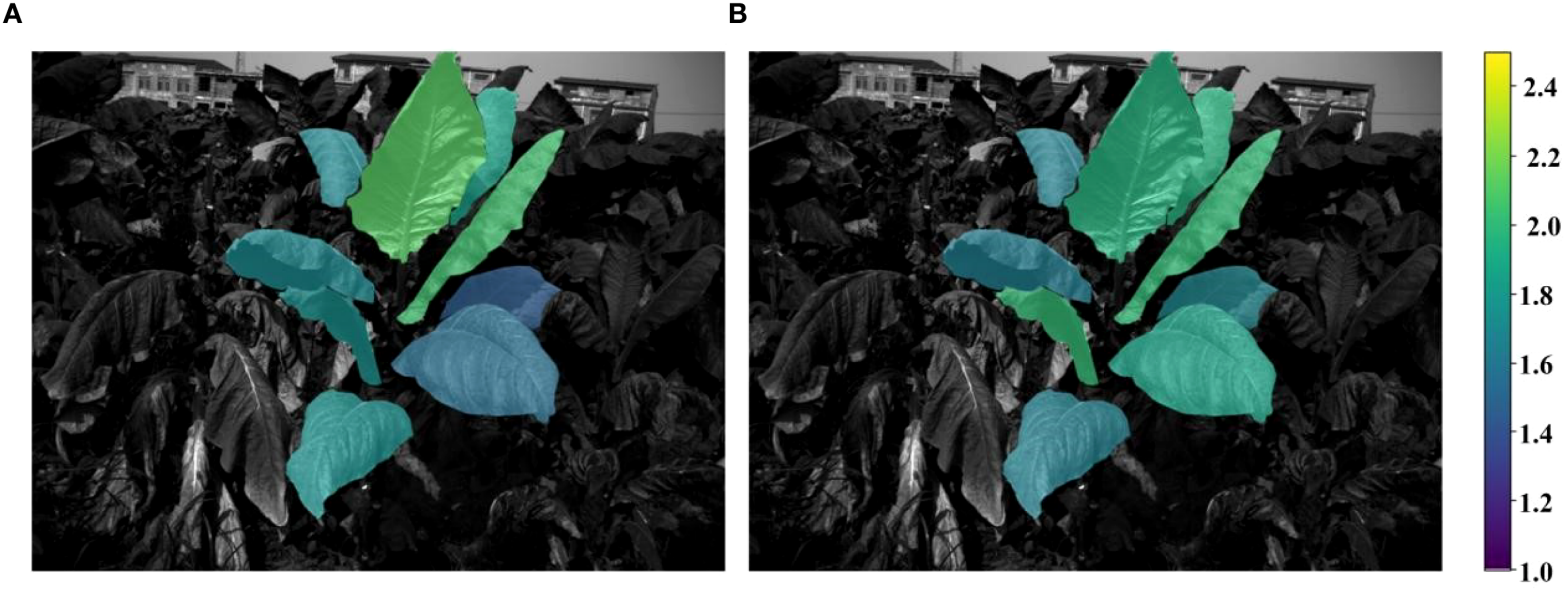
Distribution of true and predicted total nitrogen content.

**Figure 17:**
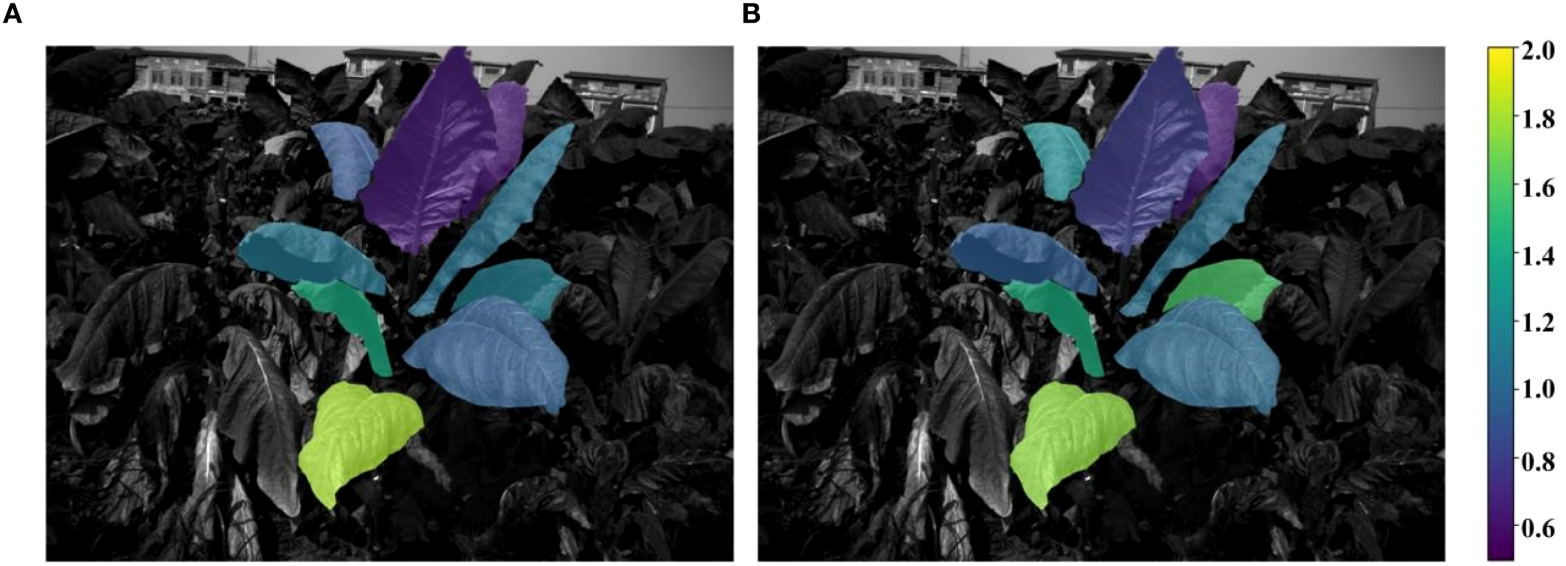
Distribution of true and predicted nicotine content.

After training the field-scale prediction model, it was applied to predict the total nitrogen and nicotine content of each tobacco leaf in the test set. The specific process involved using the AO-YOLOv8 segmentation algorithm to segment the tobacco leaves in the field images from the test set, obtaining the image data for each leaf’s individual region. Spectral and texture features were then extracted from the leaf images and input into the trained LSTM model for accurate prediction of the total nitrogen and nicotine content for each leaf. Once the predictions were completed, to present the results more intuitively, the predicted total nitrogen and nicotine content data were visualized. The content information was mapped onto the original field tobacco plant images using different colors. The final visualization results, as shown in **Figures 16**, **17**, clearly displayed the distribution of total nitrogen and nicotine in different parts of the tobacco leaves in the field. There were significant differences in the content of different leaf positions. The total nitrogen content gradually increased from the bottom to the top of the leaves, while the nicotine content presented the opposite trend.

## 4 Discussion

In terms of leaf segmentation, the results of this study showed that the improved AO-YOLOv8 algorithm could accurately segment tobacco leaves from multispectral images under field conditions. On the test set, the model achieved an mAP50 of 87.3%, an mAP50–95 of 67.3%, and an mIoU of 83.4%. In terms of segmentation accuracy, Zhang et al. first applied the SAM model for tobacco leaf segmentation and reported an mIoU of 83.81%, which is lower than the corresponding metrics achieved in this study (Zhang et al., 2023). Gu et al. proposed the BCMask model for instance segmentation of chrysanthemum seedlings, obtaining an mAP50 of 83.07%, which is also lower than our results (Gu et al., 2023).

In terms of chemical composition estimation methods, traditional determination of total nitrogen and nicotine in tobacco relies on destructive laboratory analyses, which, although accurate, are time-consuming and unsuitable for large-scale monitoring. Hyperspectral or multispectral imaging enables rapid and non-destructive detection (Guo et al., 2023; Tian et al., 2025). In this study, both spectral and texture features were combined to estimate total nitrogen and nicotine. The results demonstrated that texture features played an important complementary role in chemical composition estimation, which is consistent with the findings of Yang et al (Yang et al., 2025).

Under laboratory conditions, the fused dataset of spectral and texture features achieved high prediction accuracy (R² = 0.8634 for nitrogen, R² = 0.8735 for nicotine), showing clear advantages compared with methods based solely on spectral features for estimating total nitrogen (R² = 0.73) (Guo et al., 2023) and nicotine (R² = 0.797) (Tian et al., 2025). This confirms that, in addition to spectral features, the structural information of leaf surfaces expressed by texture features (such as veins, roughness, and uniformity) can serve as an effective supplement, providing a more comprehensive characterization of tobacco leaves. However, when extended to field validation, the performance decreased (R² = 0.6771 for nitrogen, R² = 0.5735 for nicotine). This decline can be explained by three factors: (i) shadow interference from overlapping leaves affecting spectral acquisition; (ii) the curved and drooping morphology of field-grown leaves reducing texture reliability; and (iii) residual segmentation errors introducing local noise. Such discrepancies between laboratory and field results are consistent with previous remote sensing studies, where canopy complexity and occlusion were shown to constrain model generalization. Comparative analysis revealed that this discrepancy primarily originated from three environmental factors: first, shadow interference caused by mutual leaf occlusion in the field directly affected the accuracy of spectral acquisition; second, compared to the full-view observation of flat leaf samples in laboratory settings, the curved and drooping morphology of tobacco leaves under natural growth conditions led to difficulties in texture feature extraction. Finally, although leaf segmentation was applied, overlapping leaves and slight segmentation inaccuracies in the field could still introduce local feature errors.

As shown in **Figure 17**, in the visualization of the spatial distribution of nicotine prediction, the magnitude of nicotine content differed among canopy (lower part> middle part > upper part), which was consistent with the previous research results (Fan et al., 2016; Zhang et al., 2006; Li et al., 2005). In the early growth stage of tobacco, the nicotine content of lower part was higher than middle part, followed by upper parts. After topper, a large amount of nicotine was synthesized and mainly transported and accumulated in the upper leaves, and the nicotine content of upper part was higher than middle part, followed by lower part.

Compared with traditional spectral analysis methods, this study has achieved innovative breakthroughs in two dimensions: data processing and model construction: (i) Effectively compensating for the deficiency of spectral information through texture parameters. (ii) Four deep learning architectures such as LSTM were introduced. The nonlinear activation function among them can capture the complex interaction between spectral reflectance and texture features, thereby improving the accuracy of the final prediction.

## 5 Conclusion

Aiming to enhance the accuracy of estimating total nitrogen and nicotine content for tobacco plants in field, this study proposed an innovative approach that improved leaf instance segmentation method and integrated spectral and texture data based on deep learning algorithm. The AO-YOLOv8 improved by C2f_AA and OREPAGELAN achieved higher segmentation accuracy at leaf and canopy scales compared to several methods, which effectively distinguished leaf boundaries and overlapping regions. The fusion method of multispectral and texture features established by LSTM network achieved optimal prediction accuracy for total nitrogen and nicotine at the laboratory leaf scale, with coefficients of determination (R²) of 0.8634 and 0.8735, respectively. The estimation model results of the LSTM at the plant scale in field showed coefficients of determination (R²) of 0.6771 for total nitrogen and 0.5735 for nicotine. The proposed method enables spatial visualization and accurate measurement of nitrogen and nicotine distribution across the plant, offering a cost-effective and non-destructive solution for tobacco quality monitoring and production control.

## Data Availability

The raw data supporting the conclusions of this article will be made available by the authors, without undue reservation.
